# Vampire Problems in Pediatric Kidney Transplantation‐Navigating Transformative Experiences and Informed Consent

**DOI:** 10.1111/petr.70477

**Published:** 2026-09-30

**Authors:** Vanessa Thiel, Aviva Goldberg

**Affiliations:** ^1^ McGaw Medical Center Northwestern University Chicago Illinois USA; ^2^ Ann & Robert H Lurie Children's Hospital of Chicago Chicago Illinois USA; ^3^ Max Rady College of Medicine University of Manitoba Winnipeg MB Canada

## Abstract

There are clear legal and ethical requirements for informed consent in pediatric kidney transplantation. While much is done pre‐transplant to educate patients and families on the risks and benefits of transplantation and its associated medical therapies, it can be difficult to truly prepare them for the transformative nature of the transplant‐ how it will change their lives, their families' lives, and their view of themselves and their world. Using the lens of “transformative experiences” as described by philosopher L.A. Paul, we describe the transformative nature of pediatric kidney transplantation. We encourage the health care team to acknowledge this transformative experience, and outline practical strategies including shared decision making and patient and family engagement to help communicate with families undergoing this transformation, both before and after pediatric kidney transplantation.

**Abbreviations**
KT: kidney transplant
PFE: patient and family engagement
SDM: shared decision making

## 1 Information Sharing and Decision Making in Pediatric Renal Transplant

Kidney transplant offers significant survival and quality of life advantages compared to dialysis, and remains the preferred treatment modality for children with end stage kidney disease [1, 2]. Decision making regarding transplant however, is complex and multifactorial. Patients and families considering transplant are provided with an abundance of information regarding pre‐transplant testing, donor type, wait‐listing, the surgical procedure and peri‐transplant care [3]. Families must also consider and temper expectations for post‐transplant life, and understand the continued need for intensive medical management and the possibility of post‐transplant complications such as rejection, malignancy, disease recurrence and need for re‐transplantation, among others [4]. All things considered, families of children eligible for kidney transplant have an enormous amount of information to process and a multitude of decisions to make. While the information is both necessary and valued, patients acknowledge that it can also be overwhelming [5]. Information processing is often limited by the ability of the patient or family to understand and weigh the risks and outcomes, and to choose among a host of non‐ideal but necessary options. Families have expressed feeling unprepared for reality after transplant, despite the informed consent conversations that occurred pre‐transplant [6].

Further, emotional and psychological influences may have an impact on the full comprehension of risks and benefits. Children themselves may have limited decision making capacity due to their illness, its associated co‐morbidities or their developmental status. Parents are asked to make these decisions at the same time that they are caring for sick children with chronic kidney disease, with its attendant emotional distress and instability [4]. After transplant families must then navigate immediate changes in the medical complexity and needs of their child's new condition, in ways in which pre‐discharge education and training may not entirely capture. Some parents may themselves be recovering from living donor surgery, or caring for a living donor family member in addition to their recipient child. This transition can impact the overall functioning, quality of life, and mental health of the child and family, and effects may persist years after transplant [7, 8]. Consent to transplant thus involves complex decision making at many points along the transplant journey, influenced by a number of emotional social factors, and medical considerations for their child.

## 2 Ethical and Legal Obligations for Informed Consent

Informed consent in pediatric transplantation is essential as it is both a legal and ethical imperative in all of medicine. The concept centers around respect for bodily integrity and autonomy and originated in relation to surgical procedures, which were sometimes unwarranted and often performed without prior discussion. In the modern American healthcare system the requirement has now expanded to include medical investigations, treatments and procedures of all sorts, except in the most emergent situations [9]. In the 1957 case *Salgo v Leland Stanford Jr University Board of Trustees*, the plaintiff Mr. Salgo sued the Stanford University Medical Center for improper disclosure of risk related to an imaging procedure. Here the court ruled, “…in discussing the element of risk a certain amount of discretion must be employed consistent with the full disclosure of facts necessary to an informed consent” [10]. Thus, the basic legal obligations of informed consent were established and its ethical imperative as a means of beneficence and patient autonomy, was born. Later, *Canterbury v. Spence* (1972) further defined and established what information in particular is to be included in the informed consent process and shifted the relative importance of information to the “reasonable patient” standard [11, pp. 157–166]. This emphasizes the *patient's* values and preferences in medical decision making over that of the physician's assessment of what their patient should need to know, effectively highlighting the ethical imperative of patient autonomy and self‐determination [12]. While relatively straightforward for many decisions (e.g., deciding between an open or laparoscopic approach to a needed appendectomy) this standard can become quite complex when the information encompasses both surgical and medical therapies, when both short and long term sequelae need to be considered, when prognoses are unclear and when patient and caregiver values and preferences are different from the clinician's or from each other. The default is generally to give more information, not less, but more information does not necessarily lead to better decisions and can, as demonstrated in the research ethics literature, often overwhelm decision makers [13].

In pediatrics, when children often lack the capacity to make their own medical decisions, parents are then designated the surrogate decision makers to make the decision with the best possible outcome for their children. Though this assessment of best outcomes is often a reflection of parental subjective values and preferences, parents do not have the same discretion that they would have for their own medical decisions. They are charged with deciding what is best for their child in a given circumstance, not necessarily what they would choose for themselves in a similar circumstance. They must be provided with sufficient information to make these decisions, as in adult decision making. However, information sharing in these situations can be complex, not only because of the complexity of the medical information itself, but because of unclear prognoses for many conditions and the emotional complexities of caring and making decisions for sick children.

While informed consent is both ethically desired and legally required, should we look differently at what we are trying to achieve when seeking informed consent for transplantation? Are there methods to properly prepare families for this journey while acknowledging how much we do not know and cannot predict?

## 3 The Transplant Experience

In addition to the specific medical, social and psychological considerations of transplant, the entire experience itself is *transformative*, as described by patients and families. “…But maybe one or two weeks after the operation, the transformation in X (the child), it was phenomenal”, one parent stated in a qualitative study that described how overall, kidney transplant was considered a turning point which led to transformation at the physical, mental, emotional and social level [14]. Another qualitative study investigating patients' “kidney identities” revealed several themes relating to the transformation of personal identity in transplant patients and caregivers [15].

Even when a patient or family does not utilize “transformational” terminology, described experiences of patients and families after renal transplant can be interpreted as that of a transformative experience. Families and patients have described adjusting to a “new normal” with transitions to complex regimens after transplant [16]. Some adolescents have described development of a new identity, to the point of having identity crises after transplant—“I don't really know what my personality is now. I used to be really shy but now I'm really outspoken…” [17]. Other families have described having a completely new life perspective after transplant, and view transplant as a “second chance” [18].

The importance of transplant as an *experience* has been emphasized by patients and families. In a qualitative study of pediatric transplant recipients, the children themselves expressed issues with the education they had received as being focused on the transplantation *process* but not including discussions of *life* with a transplant. These patients reported feeling unprepared in their experience after transplant [18]. Studies have also identified gaps in the understanding of patients and families with their post‐transplant experience. Caregivers have been struck with how burdensome frequent post‐transplant care can be, and others have expressed not truly grasping the reality of transplant as a treatment and not a cure [18, 19].

Given the numerous medical and surgical considerations of transplant, as well as the fact that transplant itself is a transformative experience in which the effect on identity cannot be predicted, how then can we properly discharge our duty to provide adequate information to patients and families in their transplant journey? How can we help them better understand and prepare for not only the medical needs, but also for life and its experience, after transplant. How can we continue to uphold the ethical principles—non‐maleficence, beneficence, autonomy—inherent in our discussion of informed consent for transplant?

## 4 Transformative Experiences

Philosopher L.A. Paul investigates this notion of “transformative experience” in her book of the same title. She first uses the radical (and obviously fictional) example of being offered the chance to become a vampire, trading mortality for eternal youth and a craving for human blood. She posits that since the lives before and after this decision are so extremely different that it is impossible to make a purely evidence‐based decision about whether or not to accept the vampire's bite. She follows with several more realistic examples of transformative experiences such as becoming a parent or restoring hearing via cochlear implants to explain how with some experiences, it is impossible to understand the post‐experience life until one has actually gone through the experience. Each of these experiences fall into two categories: epistemically transformative and personally transformative experiences. An epistemically transformative experience is the “kind that teaches someone something they could not have learned *without* having that kind of experience” [20, p. 15]. A personally transformative experience is that “sort of experience that can *change* who you are, in the sense of radically changing your view….” [20, p. 17]. These types of experiences can “change your subjective value for what it is like to be you, and change your core preferences about what matters” [20, p. 18].

When an experience falls into both categories, Paul classifies the experience as a truly *transformative* experience. It is these transformative experiences that are philosophically important as they raise unique problems for decision making. Paul argues that standard normative decision making may not apply to such transformative experiences since there is “ignorance about what it will be like to undergo the experience and ignorance about how the experience will change you” [20, p. 26]. Rational normative decision making requires an individual to evaluate subjective outcomes of different experiences and thus choose the experience that will most likely result in the preferred outcome. Normative decision making is thus the basis for informed consent. But how is this possible when (1) we know nothing about the experience itself and (2) we acknowledge that our subjective preferences and values may change after the experience? Paul argues we must, in these circumstances “reformulate the structure of the decision model” if we are to preserve authenticity and rationality [20, p. 79].

One means of restructuring the decision is to focus on the decision itself as the choice. Paul argues that you can solely focus on the decision of trying something new, or undergoing a transformative experience itself, rather than focus on the outcomes and how they fit current preferences. In other words, “you choose the experience for the sake of discovery itself, even if this entails a future that involves stress, suffering or pain” [20, p. 80–84]. While this may be less than satisfactory for some, Paul argues that in these extraordinary cases of transformative decisions, such as becoming a vampire, becoming a parent or undergoing a cochlear implant, we must embrace the fact that epistemically and personally, we have very little to go on [20, p. 85].

## 5 Supporting Familial Decision Making Throughout a Transformative Experience

As we've highlighted previously, kidney transplant *is* a transformative experience. Despite our best efforts at information sharing and informing decisions, for the family and patient undergoing transplant, the experience itself can be both epistemically and personally transformative. Thus, normative decision making and true informed consent can be difficult to navigate. A decision maker can and should be told that transplant improves survival and quality of life, and should be given information such as risks, benefits, complications. However, unless they already have lived experience with transplant, and even then since they have not had all the possible post‐transplant journeys themselves, they cannot truly *understand* the experience until they *undergo* the experience. For Paul, with first personal decision making “the implicit assumption of decision theory that, as long as we can describe the outcomes, we have the information we need to assign the relevant values and determine our preferences” fails [20, p. 78]. As clinicians, we therefore must recognize that limiting the pre‐transplant discussion to risks and benefits alone is a disservice to our patients and their families. If we accept that transplant is a transformative experience, we must then empower, support and appropriately counsel patients and families who have entered our exam room, are preparing to undergo this transformative experience, and who we ask to trust us to join them on their journey. See Table 1 for practical examples regarding the following recommendations.

**TABLE 1:** Tips for acknowledging transplant as a transformative experience in the clinical space.

Clinical stage	Epistemic humility	Practice points	Example language
Pre transplant	Acknowledge and appreciate the transformative nature of transplant	Reflect on previous patient experiences and feedback obtained previously Acknowledge your role as an expert but reflect on the limitations of knowledge and education	“I know what it's like to be a pediatric nephrologist, and have been with many families on this journey, but I can't truly understand what it is like to be going through this as a patient/parent/family”
	Understand that medical professionals have important knowledge to impart, but cannot fully understand, explain or predict life after transplant	Share your knowledge and clinical expertise but verbalize the variability of patient experiences and the potential life changing nature of transplant	“While I have taken care of a lot of kids with kidney disease, and there is a lot of evidence to help guide us, every child's journey is different. Kidney transplant changes the lives of all children who go through this, but not always in the same ways”
	Communicate with families that transplant may be transformative	Highlight in conversations the changes one may feel pre and post transplant (“Life *may* feel very different” or “*Some* patients have expressed feeling like a whole new person.”) Outline the differences in everyday life where possible, pre and post transplant (i.e., medication regimens, fluid restrictions vs. minimums, dialysis schedules vs. post‐transplant appointments and lab draws.) Share the transformative nature and impacts on psychological and emotional health and acknowledge that these impacts are *variable* and *unique* to individuals	“Life after transplant will be very different from your life now. Some things we will be able to predict, like the fact that you will be on different medications, have less dietary restrictions and will likely feel a lot better physically. Other things are more difficult to predict, like how you will feel about telling your friends what happened when you go back to school (for example). Sometimes people feel very different about themselves”
	Appreciate where standard pre‐transplant education may fall short in preparing families for life after transplant Discuss with families the limitations and gaps of pre‐transplant education, particularly with the emotional and psychological aspects after transplant	Discuss the expected transplant course and common or expected complications but verbalize the individuality of each transplant experience Share and normalize the experiences of other families you know personally or described in the literature	“Many feel unexpected changes or emotions after transplant, and that's OK” “Having a new kidney and new health demands can be difficult to adjust to, and that's OK”
Post Transplant:	Perform patient and family “check‐ins” regarding the transformation process. Focus not just on laboratory values but also on personal, psychological changes, coping and adjustment Allow time and space to revisit pre‐transplant education and adjust communication strategies for future patients based on this feedback	Inquire about changes in routine or daily life such as changes in schedule, social life, sports/activities, education, or even diet Ask patients and families about pre/post transplant emotions and experiences Assess if there are aspects the patient/family felt unprepared for, seek feedback as to what can be done to improve preparedness	“Have you noticed any changes in yourself or with your family after transplant?” “Do you feel different in ways you expected after the transplant? In ways that you didn't expect?” “What is one thing you wish you had known before transplant?” “What advice would you give to your past self?”
Continuous:	Incorporate frameworks such as shared decision making and patient and family engagement into clinical practice Re‐evaluate values and preferences throughout the process both before and after transplant Consider incorporation of transplant‐specific social work and psychology to meet patient and family mental health needs Consider incorporation of other ways of knowing into the pre‐transplant education of pediatric kidney transplant candidate patients and families (support groups, peer‐to‐peer programs, kidney camps) Be attentive to cultural and personal values that may impact what they want to know and how they want to communicate		

First, it is essential as clinicians to acknowledge and appreciate the nature of the transformative experience itself. In Paul's example of parental decision to have their child undergo or forgo cochlear implantation, “shedding light on the transformative nature of the choice can allow us to cast doubt on how parents can be expected to rationally evaluate these arguments about the subjective value of future experience” [20, p. 47]. In other words, recognizing and respecting transplant as transformative allows us to understand where our pre‐transplant information and education can and likely will fall short in *truly* preparing families for life post‐transplant.

Second, we must acknowledge that no one can accurately predict what life will look like after transplant for a particular child and family, which requires a degree of epistemic humility that can sometimes be hard to cultivate. As transplant team members we often assume that patients expect us to know everything and to accurately predict their child's kidney health future. Despite our vast knowledge, training and expertise, however, admitting what is unknown or unknowable in a situation can actually help promote honesty and collaboration in medical decision making and signal to families that we can be trusted, both to tell them what we know and what we cannot know with certainty.

Finally, once we as health care professionals acknowledge these gaps, it is important to be open in communicating and acknowledging this information with families. Emphasizing the transformative nature of transplant can help families understand that despite extensive education pre‐transplant or pre‐discharge, there may always be elements of the unknown. Emotionally and psychologically, this can help patients and families understand that those shifts in their perspectives, values and preferences are reasonable and in some ways expected, which may help validate their emotions and experiences. Once we acknowledge, open up space, and allow for patients and families to experience transformation, we as clinicians may then have opportunities along the way to perform post‐transplant “check ins” and discuss the on‐going experience.

## 6 Opportunities to Support Familial/Patient Autonomy and Decision Making

While acknowledging transplant as transformative can be valuable and help with creating realistic expectations of this new and unknown life post‐transplant, it is important to develop and promote decision making and autonomy for patients and families living with chronic and end stage kidney disease, where options regarding when or whether to transform may be limited. It is therefore important to incorporate established frameworks and strategies which empower and support patient and families throughout the transformation. For example, a shared decision making (SDM) framework is applicable and should be utilized throughout the transplant journey [21]. SDM incorporates both the patient's values and preferences in addition to the clinician's expertise and recommendations, to come to a unified, shared decision [22]. SDM can be implemented when there is more than one medically reasonable option [23], which may often be the case in chronic kidney disease management and pediatric kidney transplant [21]. For example, although pre‐emptive transplant is medically preferable to starting dialysis, transplant timing requires consideration of family and patient input on preferences, values and goals in addition to clinician recommendations. In a case where either transplant or dialysis may be medically reasonable, SDM may be implemented to decide which renal replacement therapy is best at that moment, for that family. Other examples in which shared decision making may be implemented include decisions to seek and/or accept a living donor, whether to participate in a paired exchange program, and whether to accept a deceased donor offer from a less‐than‐ideal donor [21]. Since we've highlighted that a transformative experience may change one's own preferences and values throughout and after the experience itself, reevaluation of goals and preferences begins early, and is continuous throughout the process.

Another means of empowering patients and families throughout a transformative experience is the utilization of Patient and Family Engagement (PFE), defined as “behaviors and actions of children, caregivers, and health care professionals that support active child and caregiver participation in the care of the child and address individual family preferences to positively influence specific child outcomes” [24, 25]. The concept again relies on shared interest in clinical outcomes from both the clinician and family, with personalization and inclusion of a family's values, preferences, unique needs and circumstances. Again, PFE should continually evolve with the patient and family's needs and preferences throughout their transformative experience. Utilization of both SDM and PFE can help empower patients and families throughout their transplant journey. It can also help clinicians with the continual reassessment of values and preferences related to care, which may change significantly throughout this transformative process.

## 7 Conclusion

Transplant is a transformative experience, but standard discussions of informed consent, and risks and benefits can fall short of acknowledging this reality. Given the life‐changing magnitude of transplant, it is important for clinicians to recognize this transformative process and incorporate this recognition into clinical practice and care. Utilizing frameworks such as Shared Decision Making (SDM) and Patient and Family Engagement (PFE) can help to incorporate and continually reassess the patient and family's values and preferences, and promote both autonomy and collaboration throughout the transformative process of transplant. While discussion here is focused on the transformation of pediatric renal transplant patients, future direction of this work can and should include the journey leading up to transplantation as well as expansion to other solid organ transplants.

## Data Availability

Data sharing not applicable to this article as no datasets were generated or analysed during the current study.
